# Mortality and causes of death of traumatic spinal cord injury in Finland

**DOI:** 10.1038/s41393-024-01047-9

**Published:** 2024-10-30

**Authors:** Elina Johansson, Eerika Koskinen, Mika Helminen, Aki Vainionpää, Teemu M. Luoto

**Affiliations:** 1 Department of Neurology, Tampere University Hospital, Wellbeing Services County of Pirkanmaa, Tampere, Finland; 2 Tays Research Services, Tampere University Hospital, Wellbeing Services County of Pirkanmaa, and Faculty of Social Sciences, Health Sciences, Tampere University, Tampere, Finland; 3 Department of Rehabilitation, Seinäjoki Central Hospital, Seinäjoki, Finland; 4 Department of Neurosurgery, Tampere University Hospital, Wellbeing Services County of Pirkanmaa, and Tampere University, Tampere, Finland

**Keywords:** Spinal cord diseases, Risk factors

## Abstract

**Study design:**

Prospective cohort study.

**Objectives:**

To study the mortality rates of TSCI patients compared to matched controls and to examine possible TSCI-related mortality risk factors and causes of death.

**Setting:**

Oulu and Tampere University Hospital, Finland.

**Methods:**

All consecutive patients with a new TSCI were included in a prospective study (n = 344, 2012-16). All patients were followed until death or the end of 2019. Patients were compared to a control group formed by randomly choosing gender, age, municipality, and calendar time-matched controls (5 controls/TSCI patient). Standardized mortality ratios (SMR) were calculated using general population mortality rates. Mortality information was extracted from the Statistics of Finland (Helsinki, Finland).

**Results:**

TSCI patients had an increased mortality (SMR = 2.9) compared with the Finnish population. During the observation period, 26% of TSCI patients and 12% of the matched controls died. Of the TSCI patient deaths, 51% occurred within the first two years postinjury. Increased age, severity of TSCI (as per International SCI Core Data Set) and fall were related to mortality (p < 0.05). The two most common etiologies of death were: circulatory (30%), and pulmonary diseases (28%). Pneumonia was the single most frequent disease leading to death among TSCI patients.

**Conclusions:**

During the first years after injury, the mortality of the patients with TSCI is double compared to the controls. Most deaths occur within two years postinjury. Elderly patients with more severe fall-related injury have the highest mortality risk. Circulatory diseases and pulmonary diseases, especially pneumonia, are the foremost causes of death after TSCI.

## Introduction

Traumatic spinal cord injuries (TSCI) often lead to widespread physiological impairments and medical complications. Improvements in survival over the decades have been reported, but despite improved medical treatment and care, life expectancy remains below that of the general population [1–3]. Age at the onset of injury, level, and AIS grade of injury have been stated to be the major predictors of mortality. Causes of death among patients with TSCI have changed from urinary tract diseases to causes more comparable with the general population [2, 3].

In order to improve the care and recovery of patients with spinal cord injury (SCI) with complex medical problems, specialized SCI centers have been established internationally. Also in Finland, the acute care, subacute rehabilitation, and life-long follow-up of patients with SCI were centralized in 2011 [4]. The centralization created an opportunity to carry out epidemiological research with better case coverage regardless of the severity of injury and starting from the acute phase [4]. Prior Finnish findings on TSCI mortality are based on a study conducted before the centralization of TSCI care and covered only patients admitted to a rehabilitation center [5] excluding patients with minor motor and sensory dysfunctions not needing institutional rehabilitation and patients with barriers to rehabilitation. Also, the demographics of TSCI have changed since the former study from 1997 to 2005 [6]. The stereotypical TSCI patient is nowadays older and sustains an injury due to low-level fall with an incomplete tetraplegia [6].

The objectives of our study were to determine the mortality of the TSCI patients compared to matched controls and to examine the possible TSCI-related mortality risk factors and the causes of death.

## Methods

### Material

Since 1st of May, 2011, in Finland, the acute care, subacute rehabilitation, and life-long follow-up of patients with SCI is centralized to three university hospitals: Helsinki, Oulu, and Tampere [4]. All consecutive patients with a new TSCI admitted to two out of three SCI centers in Finland (Tampere University Hospital; January 1, 2012 - December 31, 2015 and Oulu University Hospital; May 1, 2012–April 31, 2016) were included in a prospective multicenter study [6]. In this study, all these patients were followed until death or the end of year 2019 (mean = 5.3 years). Patients were compared to a separate control group formed by randomly choosing gender, age, municipality, and calendar time-matched controls from the general population, five matched control subjects per one TSCI patient. The control group was obtained from Statistics of Finland (Helsinki, Finland) [7].

### Data collection

In both SCI centers, a consultant physician together with a multidisciplinary rehabilitation team evaluated all the patients in the acute phase as a part of a routine treatment and rehabilitation program [6]. The International Standards for Neurological Classification of Spinal Cord Injury (ISNCSCI) were used to evaluate and classify the neurological consequence of TSCI [8]. Epidemiological characteristics were collected and classified using the International SCI Core Data Set [9].

The official Causes of Death Register of Statistics Finland contains information on all deaths since 1936 in people with permanent residence in Finland. Physicians are obliged to report deaths on a standardized death certificate. The Finnish death certificates [10] contain the following information: the date of death, the underlying cause of death, the direct cause of death, the contributing conditions, the place of death, the manner of death, the method of establishing the cause of death. All the death certificate information is stored to the official Causes of Death Register of Statistics Finland. Mortality data of the patient group and the control group were obtained from Statistics of Finland. The original death certificate documents (in pdf format) of the TSCI patients were also requested. In addition to the aforementioned information, the death certificates contain a description section with details of events, diseases and circumstances leading to death.

To determine the cause of death, we collected information from the (i) official cause of death register of Statistics Finland and ii) death certificates (TSCI patients). The underlying cause of death is defined by the World Health Organization as the disease or injury which initiated the train of morbid events leading directly to death, or circumstances of the accident or violence which produced the fatal injury [11]. TSCI is often a chronic condition that is related to increased morbidity. TSCI can be directly or indirectly related to mortality with varying time delays and trains of events. The initial trauma leading to TSCI is in many cases classified as the underlying cause of death [5]. As a result, the actual last clinical event leading to death is often disregarded. To collect more detailed information on the causes of death in TSCI patients, all death certificate data were reviewed by a neurologist (E.J.) and a neurosurgeon (T.L.). The causes of death were re-coded by extracting the information from the death certificates. In cases where the initial trauma was specified as the underlying cause of death, the direct cause of death was used, if possible or the cause of death was deduced from the whole death certificate information. Only in the cases where death occurred in the acute or subacute phase, i.e. < 14 days after injury [12], the initial trauma leading to SCI was listed as the cause of death. Also, in the cases where there was a discrepancy between data from the official cause of death register and the death certificates, we deduced the cause of death from the whole information. ICD-10 (International Classification of Diseases - 10^th^ edition) was used to classify diseases causing death. Univariate tests were used to test the strength of a relationship between two variables. In multivariable analysis, the model variables were selected based on clinical relevance prior to univariate analyses. Concerning variables “AIS grade” and “severity of TSCI”, the group of “Unknown or not applicable” was included in the univariate analysis. In multivariable analysis, all AIS A patients were determined as complete injuries, and all other grades including unknown or not applicable were considered as incomplete injuries.

### Statistical analysis

Continuous variables were presented with descriptive statistics (mean = M, standard deviation = SD, median = Md, and range). The Mann-Whitney-U-test and T-test test were used to calculate the differences between patient groups. For categorial variables, the frequencies and percentages were calculated. The differences between groups were examined by the Fisher’s exact test or chi-squared test. A *p* value < 0.05 was considered statistically significant. Age- and gender-adjusted standardized mortality ratios (SMR) were calculated as the ratio of observed deaths of TSCI patients to expected deaths in the general Finnish population using Finnish Life Tables [7]. The Vandenbroucke method was used to establish 95% confidence intervals (CI). Kaplan-Meier curves were used to visualize survival, and log-rank tests were carried out to compare survival between patient and control groups. The risk indicators for mortality of TSCI patients was assessed using multivariable Cox proportional hazard regression, using gender, completeness of TSCI, neurological level, age (60 years or less, over 60 years), and etiology as predictors. SPSS version 28.0 (IBM, Armonk, NY) was used to perform all the statistical analyses.

## Results

### Population

The characteristics of the patient group (n = 344) are shown in Table 1. The mean age of the patients was 58 years, and the proportion of men was 73%. Of the patients, 69% were tetraplegic and 19% had an AIS grade A injury. Fall was the injury etiology in 62% of the cases.

**Table 1:** Characteristics of patients with traumatic spinal cord injury (TSCI) stratified by the level of injury (tetraplegia/paraplegia).

Category	Tetraplegia n = 238	Paraplegia n = 106	Total n = 344
Gender (male/female)	3.3/1 (183/55)	1.7/1 (68/38)	2.7/1 (251/93)
Age at injury Mean +-SD, Md (range)	61.5 + −17.0, 64 (2–94)	50.8 + −20.4, 51 (6-88)	58.2 + −18.7, 63 (2–94)
Injury etiology (%)			
Sports	16 (6.7%)	12 (11.3%)	28 (8.1%)
Assault	4 (1.7%)	0 (0.0%)	4 (1.2%)
Transport	48 (20.2%)	24 (22.6%)	72 (20.9%)
Fall	158 (66.4%)	54 (50.9%)	212 (61.6%)
Other traumatic cause	12 (5.0%)	16 (15.1%)	28 (8.1%)
ASIA impairment scale (%)			
A	38 (16.0%)	27 (25.5%)	65 (18.9%)
B	20 (8.4%)	13 (12.3%)	33 (9.6%)
C	27 (11.3%)	19 (17.9%)	46 (13.4%)
D	134 (56.3%)	42 (39.6%)	176 (51.2%)
E	2 (0.8%)	0 (0.0%)	2 (0.6%)
Unknown or not applicable	17 (7.1%)	5 (4.7%)	22 (6.4%)
Severity of TSCI (%)			
Ventilator-dependent			11 (3.2%)
C1-4, AIS A, B, C			57 (16.6%)
C5-8, AIS A, B, C			18 (5.2%)
T1-S5, AIS A, B, C			60 (17.4%)
All AIS D			176 (51.2%)
All AIS E			2 (0.6%)
Unknown or not applicable			20 (5.8%)

*ASIA* American Spinal Injury Association.

*TSCI* Traumatic Spinal Cord Injury.

### Deceased

**Fig. 1:**
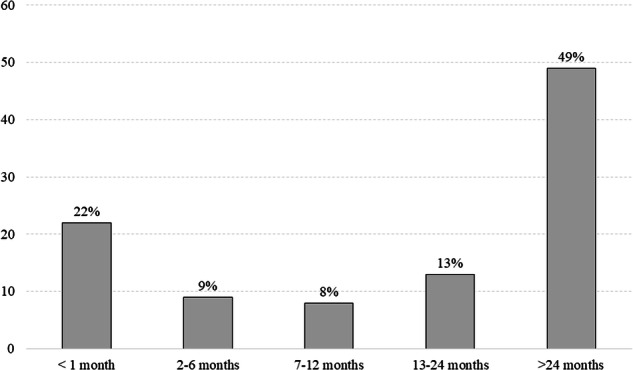
Temporal distribution (%) of deaths among patients with traumatic spinal cord injury over the study period.

During the observation period, 26% (n = 88) of TSCI patients and 12% (n = 211) of the controls died (*p* = <0.001). Table 2 presents the further characteristics of deceased individuals and survivors. The mortality rates between genders did not significantly differ. Mortality rates were higher in every age group in the patient group compared to the controls. The distribution of the delay between injury and death is shown in Fig. 1. Most deaths during this observation period occurred within two years post-injury.

**Table 2:** Characteristics of the deceased and survivors for SCI and control groups.

Category	TSCI, deceasedn = 88	TSCI, survivorsn = 256	*p-value*	Controls, deceased n = 211	Controls, survivorsn = 1508	*p-value*
Gender			1.0			0.563
Men	64 (25.5%)	187 (74.5%)		158 (12.6%)	1096 (87.4%)	
Women	24 (25.8%)	69 (74.2%)		53 (11.4%)	412 (88.6%)	
Age group			**<0.001**			**<0.001**
0–29 years	4 (9.8%)	37 (90.2%)		1 (0.5%)	204 (99.5%)	
30–59 years	9 (8.5%)	97 (91.5%)		14 (2.6%)	516 (97.4%)	
Over 60 years	75 (38.1%)	122 (61.9%)		196 (19.9%)	788 (80.1%)	

*TSCI* traumatic spinal cord injury. P values <0.05 in bold

In univariate analysis, the severity of TSCI (as per International SCI Core Data Set) (*p* = 0.000), AIS grade (*p* = 0.000) and fall as the etiology to TSCI (*p* = 0.013) were related to mortality. According to injury severity, the proportion of deaths was 55% (n = 6) in the ventilator-dependent group, 35% (n = 20) in the group of C1-C4 AIS A-C, 39% (n = 7) in the group of C5-C8 AIS A-C, 18% (n = 11) in the group of T1-S5 AIS A-C, 15% (n = 26) in the group of all AIS D, 50% (n = 1) in the group of AIS E and 85% (n = 17) in the group of unknown. Of all the patients with an AIS grade A injury, 34% (n = 22) died, and in grades AIS B, AIS C, AIS D, AIS E and unknown or not applicable 30% (n = 10), 24% (n = 11), 15% (n = 26), 50% (n = 1) and 82% (n = 18) died, respectively. Of patients who had fall as the etiology of TSCI, 30% (n = 64) died, while 18% (n = 24) of patients with other etiologies died.

### Causes of death

Table 3 presents the underlying causes of death among TSCI patients and controls according to the official cause of death register of Statistics Finland, and the cause of death based on the death certificate review. The two most common etiologies of disease-related death were circulatory diseases (n = 26, 30%) and pulmonary diseases (n = 25, 28%). Pneumonia was the single most frequent disease leading to death among TSCI patients (n = 21, 24%). In the group of TSCI patients who died within two years of the injury (n = 45), pulmonary diseases (n = 13, 29%) were more often the cause of death than circulatory diseases (n = 12, 27%). In the group of TSCI patients who died during the follow-up period after two years of injury, the most common cause of death were again circulatory diseases (n = 14, 33%) followed by pulmonary diseases (n = 12, 28%). Furthermore, during the follow-up period, among the deceased patients, pneumonia was the single most frequent disease leading to death within two years (n = 12, 27%) of injury and as well as afterwards (n = 9, 21%). Autopsy was done in 42% among the TSCI patients and in 23% among controls. Diseases were the most common manner of death (n = 79, 89%) among the TSCI patients.

**Table 3:** Causes of death of traumatic spinal cord injury patients.

Underlying cause of death (54-group classification)	TSCI Underlying cause of death (n (%))	Control Underlying cause of death (n (%))	TSCI Reviewed cause of death (n (%))
01-03 Certain infectious and parasitic diseases (A00-B99, J65)	1 (1.1%)	2 (0.9%)	3 (3.4%)
04-22 Neoplasms (C00-D48)	3 (3.4%)	58 (27.5%)	3 (3.4%)
23-24 Endocrine, nutritional and metabolic diseases (E00-E90)	2 (2.3%)	3 (1.4%)	0 (0.0%)
25 Dementia, Alzheimer’s disease (F01, F03, G30, R54)	6 (6.8%)	41 (19.4%)	6 (6.8%)
26 Other diseases of the nervous system and sense organs excl. alcohol-related	3 (3.4%)	6 (2.8%)	3 (3.4%)
27-30 Diseases of the circulatory system excl. alcohol-related (I00-I425, I427-I99)	25 (28.4%)	62 (29.4%)	26 (29.5%)
31-35 Diseases of the respiratory system (J00-J64, J66-J99)	2 (2.3%)	10 (4.7%)	25 (28.4%)
36 Diseases of the digestive system excl. alcohol-related diseases	6 (6.8%)	13 (6.2%)	5 (5.7%)
37 Diseases of the genitourinary system (N00-N99)	1 (1.1%)	0 (0.0%)	3 (3.4%)
38 Congenital malformations (Q00-Q99)	0 (0.0%)	1 (0.5%)	0 (0.0%)
39 Other diseases excl. alcohol-related	0 (0.0%)	2 (0.9%)	1 (1.1%)
40 Ill-defined and unknown causes of mortality (R96-R99)	0 (0.0%)	0 (0.0%)	1 (1.1%)
41 Alcohol-related diseases and accidental poisoning by alcohol	3 (3.4%)	8 (3.8%)	3 (3.4%)
42-49 Accidents excl. accidental poisoning by alcohol (V01-X44, X46-X59, Y10-Y15, Y85-Y86)	32 (36.4%)	4 (1.9%)	6 (6.8%)
50 Suicides (X60-X84, Y87.0)	3 (3.4%)	1 (0.5%)	3 (3.4%)
51 Assault (X85-Y09, Y87.1)	0 (0.0%)	0 (0.0%)	0 (0.0%)
52 Event of undetermined intent (Y16-Y34, Y87.2)	1 (1.1%)	0 (0.0%)	0 (0.0%)
53 Other external causes and sequelae of other external causes (Y35-Y84, Y88-Y89)	0 (0.0%)	0 (0.0%)	0 (0.0%)

*TSCI* traumatic spinal cord injury.

### Survival and determinants of death

**Fig. 2:**
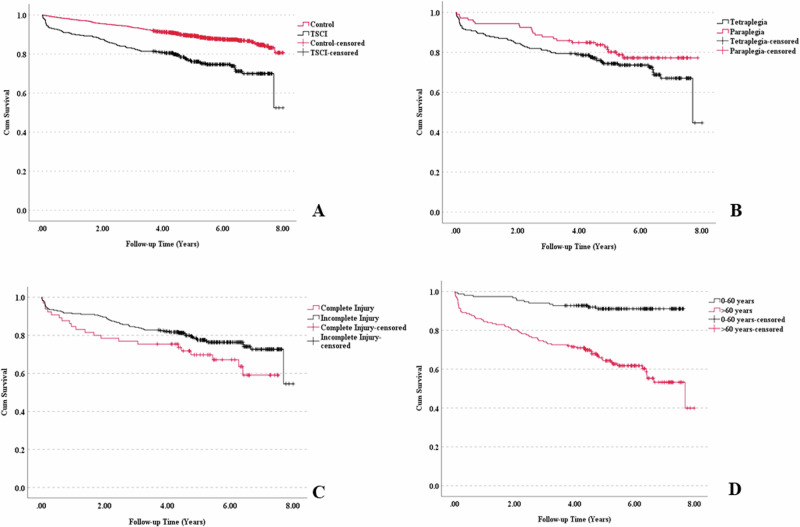
Kaplan-Meier curves. **A** = Survival of patients with TSCI versus controls (log rank p = 0.000). **B** = Survival of TSCI patients stratified to tetraplegic and paraplegic cases (log rank p = 0.138). **C** = Survival of TSCI patients stratified by the completeness of injury (log rank p = 0.067). **D** = Survival of TSCI patients stratified by the age at the time of injury (log rank p = 0.000).

The overall SMR in the TSCI group was 2.9 (95% CI 2.4–3.6). The SMR for men was 2.9 (95% CI 2.2–3.7) and for women 3.1 (95% CI 2.0–4.4). Figure 2a presents the survival curves of the TSCI patients and controls. The survival rate for the TSCI patients was 74% and for the controls 88%. Figure 2b presents the survival rates between tetraplegic and paraplegic patients. Over the study period, the survival rate for paraplegia was 79% and those with tetraplegia 72%. Figure 2c presents the survival rates for complete and incomplete injury. The survival rate for complete injury was 66% and for incomplete injury 76%. Figure 2d presents the age-stratified survival rates. The survival rate was markedly lower for TSCI patients over 60 years than for the age group 0–60 years, 61% and 91%, respectively. Cox regression model results for risk indicators for death are presented in Table 4. In this multivariate analysis, age and completeness of injury were statistically significant mortality risk factors.

**Table 4:** Cox regression model results for risk indicators for death among traumatic spinal cord injury patients.

Variable	*p*-value	Hazard ratio	95% CI
Gender	0.343		
Female		1.00 (reference)	
Male		1.265	0.778-2.057
Completeness of TSCI	0.002		
Incomplete		1.00 (reference)	
Complete		2.211	1.338-3.653
Neurological level	0.709		
Paraplegia		1.00 (reference)	
Tetraplegia		1.103	0.658-1.851
Age at onset of injury	<0.001		
≤60 years		1.00 (reference)	
>60 years		6.118	3.166-11.823
Etiology	0.880		
Sport		1.00 (reference)	
Assault	0.969	0.000	-
Transport	0.356	0.574	0.176-1.867
Fall	0.635	0.766	0.256-2.295
Other traumatic cause	0.654	0.745	0.205-2.703

*TSCI* traumatic spinal cord injury.

## Discussion

A quarter of TSCI patients died during the observation period and compared to the control group, the number of deceased was slightly more than double. Cardiovascular and pulmonary diseases were the major causes of death, pneumonia being the most prominent disease leading to death. Age at the time of injury and completeness of injury were independent predictors of death.

Previous studies of TSCI mortality, predictors of mortality, and causes of death are difficult to compare because of methodological differences regarding study populations, case selection, follow-up times, and medical certification of cause of death [1, 2, 5, 13–21]. Those studies have been conducted in different time periods in different countries and therefore with different healthcare systems, services, and policies. Many studies have focused on patients in the rehabilitation phase which excludes deaths occurring in the acute or subacute phase after injury and deaths of patients with minor TSCI-related disability or barriers to access rehabilitation [5, 15, 17, 19].

Notwithstanding the methodological differences in previous studies, improvements in survival over time and consistent predictors of mortality have been reported in several studies [1–3, 22]. Life expectancy after TSCI has improved over the decades but remains below that of the general population [1–3, 22]. A systematic review showed that overall mortality in TSCI was between 1.47 and 2.8 times higher than in the general population [3]. In line with earlier literature, TSCI was related to excess mortality in our study. Slightly more than double the amount of TSCI patients died during the observation period compared to the controls. In addition, our findings complement prior Finnish ones by Ahoniemi and colleagues, who described that the overall survival rate of TSCI was approximately half of the general population during the follow-up period 1976-2007 [5].

In earlier TSCI studies, advancements in critical care have been associated with a decline in mortality during the first two years after injury [3, 13, 19, 22]. Despite this, it still appears that a significant proportion of deaths of TSCI patients occur within 1-2 years after injury [13, 14]. Inglis et al. also recently reported that in-hospital mortality in the elderly after TSCI is high [23]. In our study, a significant number of TSCI deaths in the follow-up period occurred shortly after injury; 38% within one year and 51% within two years after injury. The 1-year mortality in the Finnish TSCI population was only slightly lower than in Estonian and Australian studies, where also deaths in the acute phase were included in the study [13, 14]. Sabre et al. showed that almost half of the deaths during the observation period 1997-2011 occurred during the first year after injury [14]. Similarly, O´Connor et al. reported that half of the deaths occurred in the first year after injury, with nearly two-thirds of these deaths occurring within the first two months [13]. On the other hand, in a study from another Nordic country, Norway, the 1-year mortality was lower than in our study, 21% [16].

Regarding the predictors of mortality, our study is in accordance with several other studies; the mortality was most strongly affected by age at the time of injury and completeness of injury, also regardless of the level of injury [1–3, 13, 14, 17, 18, 24, 25]. Also, a history of other medical conditions has previously been reported as a predictor of mortality [15, 17]. Recent studies have shown an increasing trend in the age at the time of TSCI, the proportion of cervical injuries and injuries caused by falls [6, 26, 27]. In Finland, especially low-level fall is the most dominant etiology of TSCI in patients over 60 years [6]. Moreover, in Finland, both the incidence and the average age sustaining a fatal cervical spine injury, associated with SCI in 83%, has increased markedly during the last decades [28]. Fall-induced accidents among elderly males were the most prominently increasing subpopulation [28]. In our current study, one-third of patients who had a fall as the etiology of TSCI died during the observation period. The information on the fall height (low-level fall vs. high-level fall) was not available. Also, over one-third of patients with a grade AIS A-C cervical injury died by the end of the follow-up period. It has been assumed, that nowadays an increasing number of less healthy and functionally less capable individuals are surviving to an older age in Finland and they are also more prone to falls and injuries [29]. Probably the greater occurrence of comorbidities among frail elderly contributes also to the increased risk of death in the group of patients with falls as the etiology of TSCI. Elderly males are the most prominent subgroup of individuals who sustain a TSCI, and the cause of injury is often a fall [6]. On the other hand, the incidence of TSCI among women is more evenly distributed in relation to age [6]. This baseline characteristic difference between male and female TSCI patients would allude to a higher mortality rate among males. A bit surprisingly, there was no difference in mortality between men and women in our study. In contrast, higher female mortality has been reported in four Scandinavian studies [5, 16, 17, 19], whereas large studies from the USA and UK have shown the opposite [1, 2]. Furthermore, ventilator-dependency has been stated to have a strong effect on mortality [1, 2, 23]. In line with this, most of the ventilator patients died in our patient group.

Until the 1970s, renal failure and other urinary tract complications were reported to be the leading causes of death among patients with SCI [21]. More recently, probably in connection with the advancements of SCI care, the causes of death among TSCI patients have become comparable to that of the general population [21]. Nowadays the leading causes of death among patients with TSCI are pulmonary diseases, especially pneumonia, and circulatory diseases followed by infections, and suicide [2, 3, 5, 14, 16, 30]. Consistent with other earlier results, the two most common causes of death among TSCI patients were circulatory and pulmonary diseases in our study. Circulatory disease mortality of TSCI patients is comparable to the general Finnish population in which circulatory diseases cause a third of the deaths [7]. Pulmonary disease-related mortality is ten times higher in the TSCI patients than in the general Finnish population wherein the mortality due to pulmonary diseases is only about 3% [7]. In our study, pneumonia was clearly the most noticeable single diagnosis leading to death. Depending on the degree of injury, a cervical or upper thoracic SCI markedly reduces respiratory function by causing paralysis of the respiratory muscles, and disturbed balance of autonomic nervous system with a preponderance of the parasympathetic nervous system. These changes lead to reduced ability to breathe and cough effectively as well as increased bronchial secretion and constriction favoring the development of pulmonary complications like pneumonia [31]. In addition, dysphagia with aspiration is relatively common among patients with cervical SCI potentially elevating the risk for pneumonia and other respiratory complications [32].

### Strengths and limitations

Our study gives a reliable population-based estimate of the TSCI-associated excess mortality. In our study, all patients with a new TSCI were enrolled and evaluated according to the International SCI Core Data Set already in the acute phase. This enables good case coverage with generalizable results. Additionally, the detailed review of all the available death certificate data improves the accuracy of the results, while accepting the underlying cause of death would have given more prominence to the external cause of injury-producing event than to the actual cause of death. As a limitation, the SCI center of Helsinki UH was missing from our study. It is possible that patients who are residents of Tampere UHs’ or Oulu UHs’ primary referral areas but were injured elsewhere are missing from the data. The case coverage is less certain in Turku and Kuopio UHs’ primary referral areas because the acute care and rehabilitation of those patients may partially have taken place in their own and Helsinki UH’s hospital districts. Prehospital deaths were not included in our study. Autopsies were not performed on all the deceased patients, and some of the causes of death might be incorrect.

In the future, further research with a longer follow-up period is necessary to evaluate the short-term and long-term mortality and causes of death. In future studies, concerning causes of death of individuals with TSCI, it is important to take into account that, the cause of death of TSCI patients is possibly described by the index injury [5, 13]. To avoid reporting these misleadingly high external causes of death, it is crucial to use all the detailed data including death certificates.

## Conclusions

Compared to matched general population controls, slightly more than twice as many TSCI patients died during the observation period and the majority of deaths occurred within two years after the injury. Older age at the time of injury and the completeness of injury were associated with excess mortality. Cardiovascular diseases and unlike in the general population, respiratory diseases, especially pneumonia, were the most common cause of death among TSCI patients. To reduce TSCI mortality, more attention should be paid to recognition, prevention and rapid initiation of treatment and rehabilitation of respiratory dysfunction, dysphagia, and pulmonary infections. Moreover, fall prevention of the elderly has a central role in TSCI prevention.

## Data Availability

The datasets analyzed during the current study are available from the corresponding author on reasonable request.
